# Challenges in microbial ecology: building predictive understanding of community function and dynamics

**DOI:** 10.1038/ismej.2016.45

**Published:** 2016-03-29

**Authors:** Stefanie Widder, Rosalind J Allen, Thomas Pfeiffer, Thomas P Curtis, Carsten Wiuf, William T Sloan, Otto X Cordero, Sam P Brown, Babak Momeni, Wenying Shou, Helen Kettle, Harry J Flint, Andreas F Haas, Béatrice Laroche, Jan-Ulrich Kreft, Paul B Rainey, Shiri Freilich, Stefan Schuster, Kim Milferstedt, Jan R van der Meer, Tobias Groβkopf, Jef Huisman, Andrew Free, Cristian Picioreanu, Christopher Quince, Isaac Klapper, Simon Labarthe, Barth F Smets, Harris Wang, Isaac Newton Institute Fellows, Orkun S Soyer

**Affiliations:** 1 CUBE, Department of Microbiology and Ecosystem Science, University of Vienna, Vienna, Austria; 2 SUPA, School of Physics and Astronomy, University of Edinburgh, Edinburgh, UK; 3 New Zealand Institute for Advanced Study, Massey University, Auckland, New Zealand; 4 School of Civil Engineering and Geosciences, Newcastle University, Newcastle upon Tyne, UK; 5 Department of Mathematical Sciences, University of Copenhagen, Copenhagen, Denmark; 6 Infrastructure and Environment Research Division, School of Engineering, University of Glasgow, Glasgow, UK; 7 Department of Civil and Environmental Engineering, Massachusetts Institute of Technology, Cambridge, MA, USA; 8 Centre for Immunity, Infection and Evolution, School of Biological Sciences, University of Edinburgh, Edinburgh, UK; 9 Department of Biology, Boston College, Chestnut Hill, MA, USA; 10 Division of Basic Sciences, Fred Hutchinson Cancer Research Center, Seattle, WA, USA; 11 Biomathematics and Statistics Scotland, Edinburgh, UK; 12 Rowett Institute of Nutrition and Health, University of Aberdeen, Aberdeen, UK; 13 Biology Department, San Diego State University, San Diego, CA, USA; 14 Département de Mathématiques Informatiques Appliquées, INRA, Jouy-en-Josas, France; 15 School of Biosciences, University of Birmingham, Birmingham, UK; 16 Newe Ya'ar Research Center, Agricultural Research Organization, Ramat Yishay, Israel; 17 Department of Bioinformatics, Friedrich-Schiller-University Jena, Jena, Germany; 18 INRA, UR0050, Laboratoire de Biotechnologie de l'Environnement, Narbonne, France; 19 Department of Fundamental Microbiology, Université de Lausanne, Lausanne, Switzerland; 20 School of Life Sciences, The University of Warwick, Coventry, UK; 21 Department of Aquatic Microbiology, University of Amsterdam, Amsterdam, The Netherlands; 22 Institute of Quantitative Biology, Biochemistry and Biotechnology, School of Biological Science, University of Edinburgh, Edinburgh, UK; 23 Department of Biotechnology, Delft University of Technology, Delft, The Netherlands; 24 Warwick Medical School, University of Warwick, Coventry, UK; 25 Department of Mathematics, Temple University, Philadelphia, PA, USA; 26 Department of Environmental Engineering, Technical University of Denmark, Kongens Lyngby, Denmark; 27 Department of Systems Biology, Columbia University, New York, NY, USA

## Abstract

The importance of microbial communities (MCs) cannot be overstated. MCs underpin the biogeochemical cycles of the earth's soil, oceans and the atmosphere, and perform ecosystem functions that impact plants, animals and humans. Yet our ability to predict and manage the function of these highly complex, dynamically changing communities is limited. Building predictive models that link MC composition to function is a key emerging challenge in microbial ecology. Here, we argue that addressing this challenge requires close coordination of experimental data collection and method development with mathematical model building. We discuss specific examples where model–experiment integration has already resulted in important insights into MC function and structure. We also highlight key research questions that still demand better integration of experiments and models. We argue that such integration is needed to achieve significant progress in our understanding of MC dynamics and function, and we make specific practical suggestions as to how this could be achieved.

## Introduction

Microbes exist in complex, highly diverse and highly dynamic communities (Flint *et al.*, 2007; Johnson *et al.*, 2015). These microbial communities (MCs) have crucial roles in global climate regulation, human health and industrial biotechnology. Understanding, predicting and controlling MCs thus holds the key to a wealth of potential applications, from smart waste treatment plants, through probiotic treatments of gut-related diseases, to cheese and wine making (Shong *et al.*, 2012; Johnson *et al.*, 2015; Wolfe and Dutton, 2015). The need to predict MC dynamics has stimulated the development of ‘black box' mathematical models, in which microbial population dynamics is represented by global empirical functions that do not attempt to address the inner workings of the MC. This approach has proved useful in fields ranging from food science to climate modelling and wastewater treatment (Orhon and Artan, 1994; Baranyi and Tamplin, 2004). However, such models do not aim to provide mechanistic insight, and are necessarily limited by the data sets to which they are fitted.

**Figure 1:**
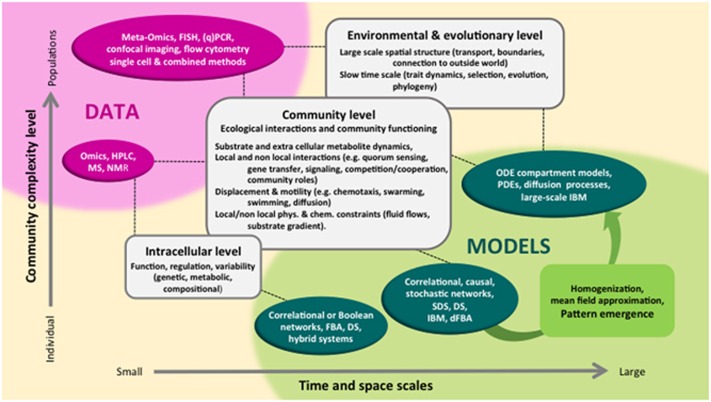
Linking MC research questions with data and modelling. Research areas, plotted according to their complexity and temporal or spatial scale, form the link between data of different forms (magenta) and modelling formalisms (dark green). Pattern emergence (light green), that is, collective behaviour obtained by up-scaling from individual description to population level, can be predicted by modelling and tested experimentally. Abbreviations: DS**,** dynamical systems of deterministic, mechanistic nature, implemented as discrete or continuous time models (difference equations, ODEs); (d) FBA, (dynamic) flux balance analysis; SDS, stochastic dynamical systems, such as Markov chains, random walks, birth–death processes; IBM, individual-based models; PDE, partial differential equations, these are deterministic structured population models (for example, according to space or traits); diffusion processes, probabilistic counterparts of ODEs and PDEs.

High-throughput sequencing, proteomics and metabolomics now allow us to catalogue the diversity of MCs to an unprecedented level of detail. These data represent a relatively unbiased compositional snapshot of the species, genes, metabolites and activities that are present in a given MC. The key challenge now is to convert this empirical knowledge into fundamental insights and testable predictions, which can be used to improve MC function for useful purposes. Here, we argue that addressing this challenge will require the development of mathematical models with a basis in mechanistic understanding, integrated with controlled experiments (Figure 1). In our view, this integration between theory and experiments is a crucial ‘missing link' in current microbial ecology. Making this link is a key step on the way to discovering possible design principles of MC community assembly and function.

## MCs as complex, interacting dynamical systems

The dynamics of MCs are driven by a multitude of interactions between their constituent microbial populations, as well as by environmental and host factors, such as immunological processes in gut microbiota or nutrient limitation in plant MCs (Klitgord and Segrè, 2010; Vorholt, 2012; Coyte *et al.*, 2015). Species interactions within MCs can be metabolic, physical, regulatory and/or signalling based, and they can drive both temporal changes in MC composition and function, and spatial organisation. Phenomenologically, an interaction between two microbial populations can be defined as the dependence of one population's growth or survival on the abundance of the other population. These interactions can be negative or positive, implying growth inhibition or facilitation. Negative interactions can arise from competition for resources such as electron donors and acceptors, nutrients, light or physical space (Hibbing *et al.*, 2010), or via direct microbe–microbe interactions, or secretion of toxins or other inhibitory compounds (Riley and Wertz, 2002). Negative interactions have been observed in evolution and competition experiments with single or several species (Passarge *et al.*, 2006; Brown *et al.*, 2008), and are also widespread among different species in natural populations, such as soil and marine bacteria (Vetsigian *et al.*, 2011; Cordero *et al.*, 2012b). Positive interactions between microbial populations can occur via several mechanisms. From a metabolic standpoint, cross-feeding of metabolic by-products, in which one population benefits from the excreted metabolites of another, is a key mediator of positive interactions (Sieber *et al.*, 2012); this also reduces the degree to which populations compete for resources (Kosaka *et al.*, 2008). Other mechanisms include production of ‘public goods' such as iron-scavenging molecules, which can be used not only by the producer population but also by others (West *et al.*, 2006).

**Figure 2:**
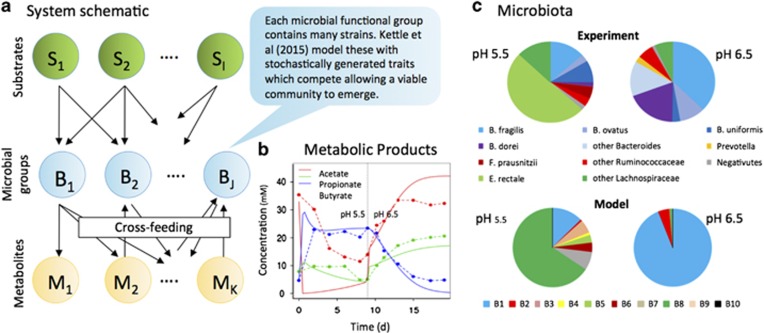
(**a**) The human large intestine and the rumen and caecum in herbivorous animals harbour dense MCs dominated by anaerobic microbes that cross-feed metabolites extensively. In recent models, these are approximated by a small number of functional groups (Muñoz-Tamayo *et al.*, 2010; Kettle *et al.*, 2015). (**b**) Comparison of this model to a fermenter experiment with a pH shift from 5.5. to 6.5 after 9 days for metabolic products (dashed lines are experiment data, solid lines are model results). (**c**) Comparison of temporal species dynamics between model predictions and data. The experimental data consists of phylogenetic groups (16S rRNA gene sequencing), simulations refer to functional groups; approximate correspondence between the two is indicated by colour coding (for example, *Lachnospiraceae* equivalent to B5 and B8, shown in green, *Bacteroides* belonging to B1 shown in blue and purple).

**Figure 3:**
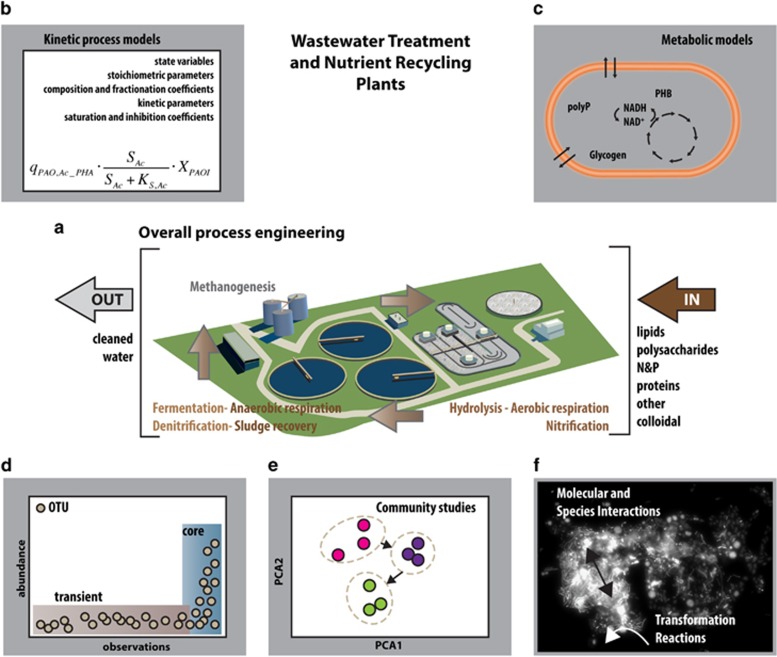
MC analysis and predictive modelling in wastewater treatment and nutrient recycling facilities (WWTP). (**a**) Carbon and nutrients are eliminated from wastewater by (micro-)biological activities before discharging the cleaned water. WWTP processes are optimised through controlling MC conditions (for example, anaerobic, aerobic) and partial recycling of the MC biomass. (**b**) Overall system behaviour and MC biomass are predicted using growth kinetic and dynamic models. (**c**) Functional metabolic models are derived from pure culture physiology or metagenomic data. (**d**) Although the WWTP system is largely engineered, the MCs form mostly spontaneous, consisting of ‘core' assemblages and ‘passenger' groups as a function of constant wastewater input, after (Saunders *et al.*, 2016). (**e**) The assemblage process depends on taxonomic relatedness and ecological interactions, and can lead to a drift of MCs over time. (**f**) MCs in WWTP occur mostly in flocs and or biofilms (picture courtesy of J van der Meer, University of Lausanne) that display immense microdiversity. Both, interactions and spatial organisation are factors that generate niches, gradients and foster co-existence.

Identification and measurement of interactions within MCs, and accurate representation of these in theoretical models, is an important basis for building understanding, and thus presents a key challenge in microbial ecology. A second challenge is the converse: to use theoretical approaches to predict species interactions in MCs from proximal data such as taxon abundances. Both cases require direct integration of theory and data in specific ways. Although many advances have been made toward such integration (for examples, see Figures 2 and 3), a number of technical obstacles remain. In the following, we discuss key areas where integration theory and data can be highly productive. We then highlight a number of broader scientific questions, which we see as crucial for the longer-term development of models for MC structure, function and dynamics. Finally, we call for development of model systems where well-controlled experiments interrogating function–structure relation in communities can be more readily performed and information and methods among multiple laboratories can be shared.

## Integrating theory and data to understand MC dynamics

### Measuring population dynamics in MCs

A key objective of theoretical models for MC dynamics is to reproduce temporal trajectories of the populations within the community. However, state of the art high-throughput sequencing generates a snapshot of the *relative* abundances of taxa or genes within a MC; it does not provide *absolute* abundance information. Furthermore, there can be inherent biases even in this relative abundance data. These limitations mean that high-throughput sequencing on its own is not sufficient to track temporal and spatial population dynamics. Complementary experimental approaches such as quantitative PCR, flow cytometry, species-specific fluorescence *in situ* hybridisation or novel combinations of single-cell and functional-targeting methods with genomics (Berry *et al.*, 2015) do yield information on absolute abundances. Yet, this information is limited, because these approaches are usually targeted, that is, need *a priori* knowledge of which populations or functions are to be investigated. Although some of these approaches can be used in an untargeted manner, for example, by using general primers for fluorescence *in situ* hybridisation or by combining single-cell separation with genome sequencing, this involves technical challenges. Up-scaling these absolute abundance measurements to the community level is an important goal. We note that combining high-throughput sequencing with quantitative PCR analysis to measure total bacterial abundance should, in principle, allow absolute taxon abundances to be computed. Yet, we are not aware of studies that have used this approach to date.

We also note that the potential of sequencing approaches to predict aggregate community function is currently limited, because neither metagenomic data nor 16S RNA gene sequence data are a perfect predictor of metabolic function for an individual species. This is an area where new sequencing and bioinformatics approaches can result in significant improvement.

### Inferring species interactions from proximal data

Direct measurement of interactions between species within a community (for example, for metabolic interactions, by tracking radioactively or isotopically labelled compounds) is an excellent way to collect the basic information needed for model building, but it is necessarily targeted to specific types of interaction. To obtain community-wide information on microbial interactions where direct evidence is lacking or restricted, one can use statistical inference based on correlations between taxon abundances from high-throughput sequence data (Fuhrman, 2009; Freilich *et al.*, 2010; Faust *et al.*, 2012). The resulting co-occurrence interaction networks can be used to make ecological predictions, such as the existence of metabolic dependencies, the fragility of communities to environmental change or the likely identity of keystone species that stabilise the community (Faust *et al.*, 2012; Berry and Widder, 2014; Widder *et al.*, 2014). Although correlational approaches can point to previously unknown interactions, they require confirmation by direct evidence (Friedman and Alm, 2012), because correlations in taxon abundances can be confounded by biases in 16S data (Zhou *et al.*, 2011) and can also arise from indirect species interactions. Examples of such indirect interactions include apparent competition by shared pathogens or predators, or the immunological response of a shared host (Brown *et al.*, 2008; McNally *et al.*, 2014). As these indirect interactions typically occur on larger spatial scales than direct interactions, development of spatially resolved methods for interrogating MCs can be highly beneficial. Existing methods such as fluorescence *in situ* hybridisation provide proximity data among species, but are invasive. Recent advances in non-invasive spatially resolved methods, such as in-line digital microscopy, for gathering population dynamics data are thus of great interest (Frentz *et al.*, 2010).

### Predicting species interactions using stoichiometric models

Many of the important interactions in MCs are mediated by metabolite exchange; thus computational approaches modelling metabolic fluxes between organisms can be used to predict interactions. These approaches are based on knowledge of the genome, which is used to build a ‘stoichiometric model' for the complete set of metabolic reactions associated with a given organism (Schilling *et al.*, 1999). Classical flux balance analysis (FBA) (Orth *et al.*, 2010) can then be used to predict the organism's metabolic fluxes. Up-scaling this approach to construct community-wide stoichiometric models or performing detailed metabolic studies of individual participants within a community (Freilich *et al.*, 2011) allows for insights into metabolic interactions occurring within a MC (Stolyar *et al.*, 2007). For example, metabolic studies have shed light on the succession of primary and secondary degraders that ensures communal accessibility of complex carbohydrates in the mammalian gut (Muñoz-Tamayo *et al.*, 2010; Eilam *et al.*, 2014; Kettle *et al.*, 2015) (Figure 2). Moreover, dynamical FBA can also form a basis for dynamical models of MCs (Mahadevan *et al.*, 2002). In these models, microbial growth is coupled to a dynamically changing chemical environment, which is in turn influenced by metabolism, as computed by FBA. Recently, such dynamical models have also been extended to include spatial resolution of community structure (Harcombe *et al.*, 2014). These developments are very promising, but it is important to note that the application of FBA to MCs is challenging. In particular, standardised methods are needed to generate reliable stoichiometric models of the large number of species involved in MCs and to be able to integrate models so that they can be simulated under a single, shared environment.

### Kinetic models for community dynamics

Kinetic models, such as the widely used Monod equation, aim to predict microbial growth rate, given the concentrations of essential nutrients and/or inhibitory chemicals, and species-dependent parameters such as the maximal growth rate (Figure 3). Extending this approach to the community level is attractive because it is conceptually simple, computationally tractable and provides dynamical predictions. However, its success depends on identification of the most important microbial species to include in the model, and the interactions between them. Moreover, the reliability of the approach depends crucially on the underlying kinetic growth model and its parameters—thus large-scale measurement of kinetic growth parameters for a variety of microbial species would be extremely useful. More fundamentally, existing kinetic growth models are often ‘*ad hoc*' and may miss aspects of the growth kinetics that are important in community function, such as product inhibition. To address this problem, new kinetic models are being developed that include features like condition-dependent changes in the maximal growth rate (Bonachela *et al.*, 2011; Desmond-Le Quemener and Bouchez, 2014) and the thermodynamics of microbial metabolism (Jin *et al.*, 2013). In particular, the inclusion of thermodynamics in kinetic growth models has contributed to improved modelling of MCs, for example in anaerobic waste treatment (Gonzalez-Cabaleiro *et al.*, 2013).

## Long-term challenges for developing an understanding of structure–function relation and dynamics in MCs

### The need to include evolutionary processes

Traditionally, evolution has been ignored in ecological models, as it is assumed to occur only on long timescales. While this assumption might hold for animal and plant communities, ecological and evolutionary timescales can coincide in some MCs, because of their short generation times, large population sizes and high rates of gene transfer. Thus, models that address MC dynamics should take into account both ecology and evolution, for example, see (Post and Palkovacs, 2009). The intermixing of ecological and evolutionary dynamics is strikingly demonstrated by long-term growth experiments with *Escherichia coli* in glucose-limited chemostats. Here, genetic diversification produces two genotypes, which cross-feed; that is, it leads to a new ecological interaction. Functional diversification at the genome level is maintained by the novel ecological interaction and vice versa (Little *et al.*, 2008). As ecological interactions evolve in MCs on the same timescales as the species themselves evolve (Cordero *et al.*, 2012a; Hillesland *et al.*, 2014), the development of modelling frameworks that include evolution of species' traits and interactions should have an important role in microbial ecology. From a modelling perspective, simplified ‘toy-models' have been developed, which include ecology and evolution, for example, in (Pfeiffer *et al.*, 2001). The challenge is to make these more realistic (for example, including more aspects of cellular metabolism). From an experimental perspective, attempts are being made to follow the evolution of selected species, for example, in natural soil MCs (Gomez and Buckling, 2013); the next step is to track more species simultaneously, and to extend to other environments, such as gut communities (for example, using axenic animal systems). An alternative approach is to use defined synthetic communities, constructed from known species, in which it is possible to track the ecological and evolutionary dynamics of individual species within the community (see for example, Mee and Wang, 2012; Bodenhausen *et al.*, 2014; Faith *et al.*, 2014).

### Social evolution and bacterial strategies

An important challenge in model development is to correctly account for the complex social organisation that can occur amongst microbes and, for experimentalists, to test how frequently social interactions occur in natural settings. For example, secretion of ‘public goods' such as toxins, enzymes, metabolic co-factors or signalling molecules can lead to intricate evolutionary dynamics (Leggett *et al.*, 2014). Game theory provides a way to model complex social behaviours in mixed MCs. Here, different microbial behaviours are abstracted as simple ‘strategies'. As an example, some microbial populations produce extracellular enzymes such as cellulases that digest recalcitrant nutrients in the environment. ‘Cheater' cells do not produce these enzymes, but nevertheless benefit from their release by ‘cooperative' donor cells. Game theory maps this scenario on to a number of classic ‘games', such as Prisoner's Dilemma, the hawk-dove game or the mutual benefit game, depending on the values of the kinetic parameters (Gore *et al.*, 2009). Extending such game theoretical models to include MC spatial structure remains a challenging area, although some important advances have been made using lattice-based simulations and continuum partial differential equation approaches (Reichenbach *et al.*, 2007, 2008).

### Community assembly and historical contingency

Community assembly, or the mechanism by which a community forms, is a widely studied topic in macro-ecology, but has been relatively little addressed for MCs (Woodcock *et al.*, 2007). For some MCs it is known, however, that historical contingency—the order in which different species arrive in the community—can have a strong impact on community composition; examples include oral communities (Teles *et al.*, 2012) and gut microbiota (David *et al.*, 2014). Importantly, different patterns of species arrival can result in different long-term interaction networks within the community (Vannette and Fukami 2014). These historical contingency effects are likely to have important consequences for the engineering and control of MCs in the environment, agriculture and medicine. Future work should systematically investigate these effects experimentally for complex MCs and, concurrently, integrate them into population dynamic models. Moreover revisiting suitably designed, older experiments with new methods may also contribute to understanding temporal processes of community assembly in MCs.

### The importance of spatial structure

**Figure 4:**
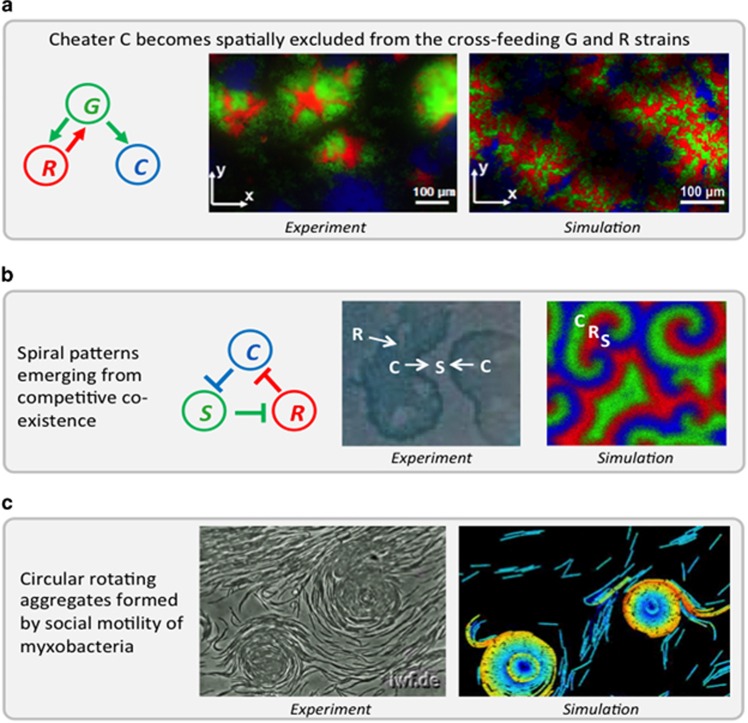
Spatial patterns can emerge from local metabolic and mechanical interactions between individual microbes. Such self-organised patterns facilitate, for example, cooperation (**a**), competitive co-existence (**b**) or formation of fruiting bodies (**c**). (**a**) Cross-feeding between G (green) and R (red) strains facilitates exclusion of cheater C (blue) over time. Cross-feeding was engineered in yeast strains and visualised by fluorescence tagging, adapted from (Momeni *et al.*, 2013). (**b**) Spiral patterns emerge from chasing in space: Colicin producer C kills sensitive S. S outcompetes resistant R, which outcompetes C in turn. Spatial structure facilitates dynamic co-existence. Experimental results on agar plates adapted from (Kerr *et al.*, 2002) and simulation of spatial system from (Szczesny *et al.*, 2014). (**c**) Experimental observation (Reichenbach *et al.*, 1968) and simulation (Janulevicius *et al.*, 2015) of circular aggregates formed by social motility of myxobacteria as an intermediate step in the development of fruiting bodies.

Another important feature of MCs is their complex spatial structure (Figure 4). Indeed, densely packed aggregates, which may be free-floating or in the form of surface-attached biofilms, are believed to be the predominant mode of life for many microbes in the natural environment, for example, MCs on marine snow particles (Kiorboe *et al.*, 2003; Elias and Banin, 2012), and are crucial in processes such as wastewater treatment (Figure 3). Within these microbial aggregates, driving factors for spatial organisation include (i) metabolite gradients caused by consumption/production, diffusion and advection, (ii) gradients of abiotic factors such as light or temperature, (iii) physical adhesion and (iv) motility. A number of well-established methods exist for modelling spatial structure development within aggregated MCs (especially biofilms). Continuum spatial models predict how microbial biomass density and chemical concentrations change in space and time and typically include diffusion, advection and mechanical forces (Klapper and Dockery, 2010). At a more detailed level, individual-based models track the location and fate of individual microbial cells within the community, taking into account a plethora of features such as spatially resolved metabolite concentrations or electrochemical interactions with a surface (for example, an electrode). The use, and further development, of such spatially explicit models is important because spatial organisation of aggregated MCs is likely to have a drastic effect on their structure and function (Momeni *et al.*, 2013). For example, biofilm infections are notoriously more resistant to antibiotic treatment than well-mixed planktonic cultures (Davies, 2003). Such improvements in modelling spatially structured MCs must go hand-in-hand with improvements in experimental interrogation of spatial structure within natural settings, and in particular development of non-invasive measurement methods.

## A call for the development of model MCs and well-controlled experiments on them

**Figure 5:**
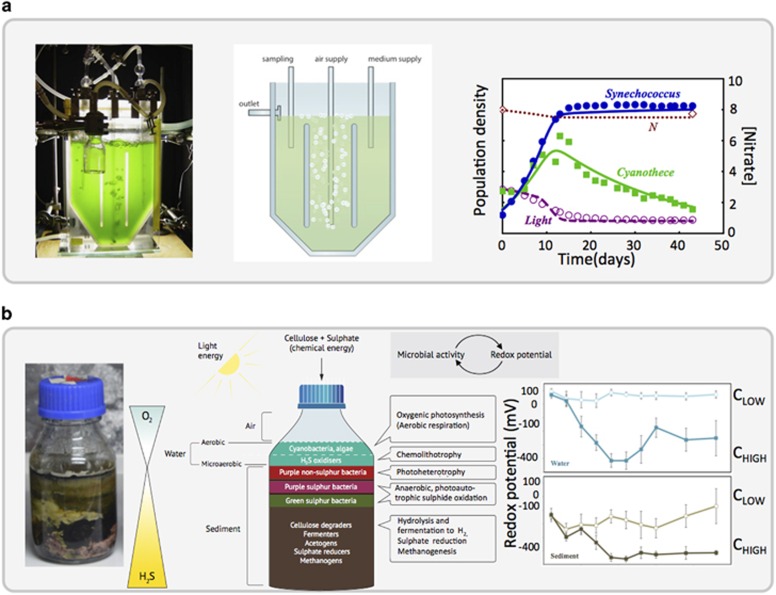
(**a**) (Left) Chemostat competition study between marine, nitrogen-fixing *Cyanothece sp.* and a non-nitrogen-fixing *Synechococcus* species (source: Department of Aquatic Microbiology, University of Amsterdam). (Middle) Schematic drawing of a chemostat. (Right) At high nitrate levels, the nitrogen-fixer (*Cyanothece*) is competitively excluded by the non-nitrogen-fixer (*Synechococcus*). Symbols are measurements; lines are model predictions (after Agawin *et al.*, 2007). (**b**) (Left) Study in Winogradsky column microcosms. (Middle) Schematic of the vertically layered structure of a mature Winogradsky column. Principal microbial types are found in different layers, their ecological activities and the associated core chemical reactions are illustrated. As a result opposing gradients of sulphide and oxygen develop. (Right) Microbial activity leads to a transient drop in redox potential in the overlying water, and a long-term drop in the sediment, at high levels of added cellulose (‘high C'). Low levels of added cellulose (‘low C') induce only a short-term reduction in redox potential.

In microbiology, fundamental understanding of microbial physiology and metabolism has been acquired by studying a set of standard microbial model organisms, for example, *Saccharomyces cerevisiae*, *Escherichia coli*, *Bacillus subtilis* and *Pseudomonas aeruginosa*, under well-defined conditions. In microbial ecology, in contrast, data collection has mainly focused on characterisation of environmental samples from a multitude of different settings. We argue here that much can be gained by focusing efforts on a more limited set of well-defined ‘model' MCs (Denef *et al.*, 2010; Groβkopf and Soyer, 2014; Estrela *et al.*, 2015), including both synthetic communities constructed from known microbial species (Bodenhausen *et al.*, 2014; Faith *et al.*, 2014; Bai *et al.*, 2015), and microcosm communities made from environmental samples (Foster and Bell, 2012; Pagaling *et al.*, 2014; Wolfe and Dutton, 2015). Such an approach offers many advantages. From a general point of view, focusing on a limited set of model systems would make results much more transferrable between studies, allowing multiple groups to work synergistically, and therefore speeding up progress toward mechanistic understanding. Lab experiments with well-defined MCs under controlled conditions, for example, in chemostats, should allow hypothesis testing via control of key external parameters such as substrate concentration, system size or temperature, and allow for multiple replicate experiments (Figure 5).

From a more specific point of view, synthetic ecological communities provide a way to limit the system to a manageable number of microbial constituents, making it simpler to analyse and model—it may even be possible to measure exhaustively kinetic and interaction parameters for an entire community, as input for theoretical models. Indeed, *de-novo* assembly of low diversity MCs (Mee and Wang, 2012) provides control of microbial interactions, non-linear effects because of adding traits or community members and evolutionary changes (Celiker and Gore, 2014; Fiegna *et al.*, 2014).

Synthetic communities can also be used to test the role of particular ecological mechanisms, such as metabolic interactions, spatial heterogeneity (van Gestel *et al.*, 2014) or induced cell death (Asally *et al.*, 2012). As a pathway toward more systematic development of synthetic model MCs, we advocate building on the success of early microbiological studies in collecting data on specific model species under standardised culture conditions. In particular, we suggest that obtaining detailed physiological data (for example, kinetic growth parameters under different conditions) on a collection of ecologically relevant microbial model organisms, would greatly facilitate the community-wide construction of synthetic MCs. Although synthetic communities do not reproduce the full diversity and complexity of natural MCs, many of the principles of community organisation and dynamics, which we learn from them should be transferable to more complex MCs.

As a stepping stone from simplified synthetic systems to natural MCs, and to address questions concerning contingency, functional redundancy, species diversity and variability and the nature of interactions in highly diverse, complex communities (Foster and Bell, 2012), microcosms provide an excellent platform (Figure 5). A microcosm consists of an environmental sample that is cultured in the lab under well-defined conditions. The microcosm often retains much of the compositional and functional diversity of the seed community, and may also have spatial structure, allowing, for example, for nutrient cycles and redox gradients (Pagaling *et al.*, 2014). As microcosm experiments can be replicated, sampled and perturbed under well-controlled conditions, they provide an excellent bridge between the simplicity of synthetic model ecosystems and the complexity of MCs in the natural environment (Pagaling *et al.*, 2014; Wolfe and Dutton, 2015). We also advocate performing experiments on a limited set of standardized microcosm communities; for example, the Winogradsky column, in which an aquatic sediment-water sample develops under lab conditions into a self-sustaining, vertically stratified, nutrient-cycling community.

## Conclusions

Despite impressive advances in our knowledge of the species composition of MCs, we are still far from achieving the level of fundamental understanding of the dynamics and function of MCs that is needed to predict and control MC behaviour. Here, we have argued that the key to achieving this level of predictive understanding is the integrative development of mathematical models with experimental data collection and method development. There are considerable challenges associated with both experimental method development and data collection, and mathematical model building in the study of MCs. These challenges are intertwined, such that tackling them effectively requires an integrated approach. To advance toward this goal, we advocate increased interaction between empirical and theoretical scientists, as well as the development of well-defined model MCs that can act as test-beds for the integrative development of experimental and modelling approaches.

How can this best be achieved in practice? Although many of the points that we make here are addressed to individual scientists, interactions between empiricists and theoreticians should be facilitated by the continuation of community-wide activities like the recent 4-month programme ‘Understanding Microbial Communities' held at the Isaac Newton for Mathematical Sciences at Cambridge University. Moreover, some of the developments, which we call for here, such as the extensive characterisation of specific model systems, can best be done by groups of scientists working together rather than by individual groups. Specific funding of such community-directed research activities, in parallel with individual research efforts, would be a very welcome development.
